# Research on calibration method of microscopic parameters of siltstone based on gray theory

**DOI:** 10.1038/s41598-023-43008-x

**Published:** 2023-09-22

**Authors:** Mengkun Wu, Ankui Hu, Shuai Zhou, Xianhui Mao, Wang Fei

**Affiliations:** 1 Key Laboratory of Fluid and Power Machinery, Ministry of Education, Xihua University, Chengdu, 610039 China; 2 Key Laboratory of Fluid Machinery and Engineering, Xihua University, Chengdu, 610039 Sichuan China; 3 School of Energy and Power Engineering, Xihua University, Chengdu, 610039 China

**Keywords:** Civil engineering, Applied mathematics

## Abstract

When the Linear Parallel Bond Model (PBM) in Particle Flow Code is used to simulate intact rocks, a basic problem is how to determine the microscopic parameters that control the macroscopic properties of the modeled rocks. After simplifying the microscopic parameters of the PBM model, this study proposes a new method of inverse performance of the regression equations of the macroscopic parameters by the gray absolute correlation combined with regression analysis, which solves the drawbacks of the current manual trial parameter calibration method to a certain extent. When this method is applied to the calibration of the microscopic parameters of the siltstone, the numerical simulation results produce good agreement between the results derived from the finite element software (ABAQUS) both in terms of macroscopic parameters and damage forms.

## Introduction

In recent years, the Particle Flow Code (PFC), developed based on the discrete unit method, has been used in a large number of geotechnical engineering numerical simulations because it overcomes the limitations of the traditional macroscopic continuity assumption and can study the macroscopic mechanical behavior of geotechnical materials from a microscopic parameters^1–6^. In geotechnical discrete element numerical simulations, the correct calibration of the microscopic parameters is the primary condition to reflect the real mechanical properties of geotechnical materials and is the key to determine the accuracy of simulation results^7–10^. At present, the selection of microscopic parameters is mainly the "trial-and-error method", i.e., a series of particle microscopic parameters are assumed to be used to simulate the sample, and the simulation results are compared with the physical experimental results of the material, and when the numerical simulation results are similar to the physical experimental results, it is considered that the proposed series of microscopic parameters can be used. However, the method is time-consuming, blind and inefficient, so a new method of microscopic parameters calibration is needed to calibrate the microscopic parameters, for which many scholars have conducted in-depth research, and more theoretical results have been obtained. For example, Wang Jinwei et al.^11^ used a combination of contour method and orthogonal test to quickly determine the microscopic parameters of rock piles by indoor triaxial drainage shear test of rock piles. Li et al.^12^ investigated the relationship between the macroscopic parameters of the flat nodal contact model by multi-factor ANOVA, calibrated the microscopic parameters by using BP (back propagation) neural network modeling and calibrated the calibration results. Zhou Xiaopang et al.^13^ used seven typical loess landslides occurred in Hefangtai from 2015 to 2017 as the research object, and found a new method of calibration of microscopic parameters applicable to landslides under such geological conditions by neural networks and other methods. Huang and Nardin^14,15^ established a qualitative relationship between microscopic and macroscopic parameters of viscous granular materials. Sanei et al.^16^ develop a new scheme to provide an initial guess for an iterative optimization method to accurately calibrate the material parameters of a strain-hardening elasto-plastic constitutive model based on test data. However, these newly proposed methods have certain disadvantages, such as complex and cumbersome processes, the need for a large number of sample data, etc., that is to say, they do not completely solve the disadvantages of the "trial and error method".

In this paper, based on the gray absolute correlation, a new method for calibrating the microscopic parameters of siltstone is proposed by using the parallel bonding model^17,18^, which is widely used in simulating geotechnical materials. The grey absolute correlation is combined with regression analysis to invert the regression equations of macroscopic and microscopic parameters, which solves the drawbacks of the current manual trial calculation parameter calibration method to a large extent. The theoretical results of this paper are of guiding significance for the calibration of microscopic parameters in discrete element numerical simulation of siltstone, and it is demonstrated that the method is applicable to the calibration of microscopic parameters in different types of lithologies and different intrinsic models, and it has a certain value of reference for the calculation of microscopic parameters of the PBM model by manual trial.

## Parallel bonding model and its microscopic parameters simplification

**Figure 1:**
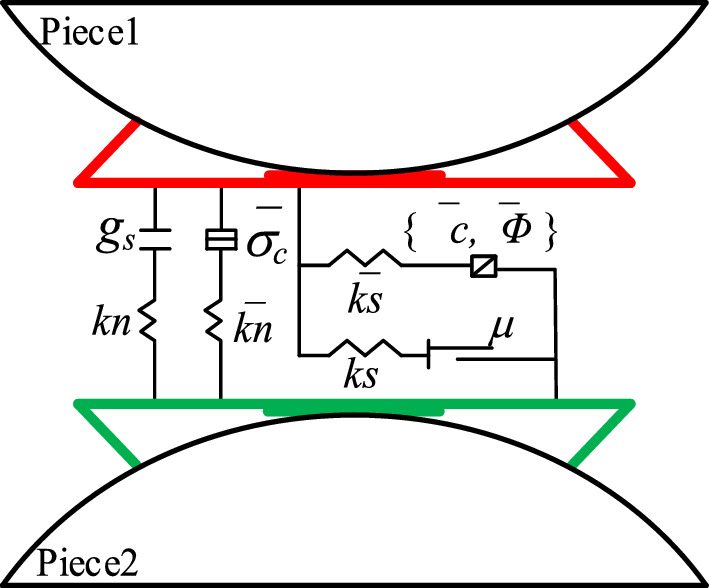
Mechanical model of parallel bond^17^.

The parallel adhesion model^2^ describes the intrinsic model with intercalated or cemented materials in finite dimensions between particles, and the relative motion of the particle contact positions can generate forces and moments, which act on the two adhesive particles, as shown in Fig. 1 in the specific mechanical model diagram. The contact force between particles consists of linear contact force ${F}^{1}$, adhesive contact force ${F}^{d}$ and parallel adhesion force $\overline{F }$; the contact moment is provided by the adhesive contact, which is divided into bending moment ${M}_{b}$ and torque ${M}_{t}$. The adhesive contact force ${F}^{d}$ can be decomposed into normal and tangential forces as follows:

1 $${F}^{d}={F}_{n}^{d}{\widehat{n}}_{c}+{F}_{s}^{d}{\widehat{s}}_{c}$$

where: ${\widehat{n}}_{c}$ and ${\widehat{s}}_{c}$ are the unit direction vectors of normal and tangential forces, ${F}_{n}^{d}$ is the normal force, and ${F}_{s}^{d}$ is the tangential force.

In the normal direction, the bonding is based on the maximum tensile stress criterion, when the maximum tensile stress σ_max_ of the bonding exceeds the tensile strength σ_t_, the bonding undergoes tensile damage; in the tangential direction, according to the Mohr–Coulomb strength criterion , when the maximum shear stress τ_max_ of the bonding is greater than the shear strength τ , the bonding undergoes shear damage. This can be seen in the following equation:

2 $$\left\{\begin{array}{c}{\upsigma }_{max}=\frac{{F}_{n}^{d}}{A}+\frac{{M}_{b}R}{I}>{\upsigma }_{t}\\ {\uptau }_{max}=\frac{{F}_{s}^{d}}{A}+\frac{{M}_{t}R}{J}>\tau \end{array}\right.$$

where: *A* is the bonding area, *I* and *J* are the moments of inertia and extreme moments of inertia of the bonding bonds, respectively, and *R* is the bonding radius.

**Figure 2:**
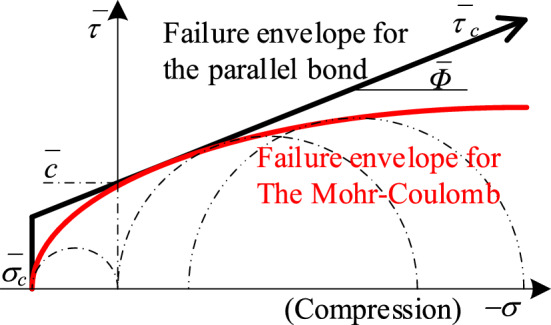
Parallel bond model (black) and Mohr–Coulomb strength curve (red).

In this study, PBM is chosen as the constitutive model for the calibration of microscopic parameters because it has the deformation characteristics of flexural and torsional resistance, as well as having the strength characteristics that are consistent with rock damage, and the specific strength curves are shown in Fig. 2.

The PBM model parameters are shown in Table 1 for a total of 13. In order to study the nonlinear mapping relationship between the microscopic parameters and the macroscopic parameters (elastic modulus *E*, Poisson's ratio ν, compressive strength σ_c_, tensile strength σ_t_), it is necessary to optimize and simplify the microscopic parameters of the PBM model to some extent. The reason is that the PBM model has a large number of microscopic parameters, which increases the difficulty of calibrating the microscopic parameters. The density ρ, the friction angle φ and the porosity *n* are parameters obtained on the basis of physical experiments, so they are not taken into account.

**Table 1:** Mesoscopic parameters of the parallel bonding model.

Symbol	Meaning	Value	Remark
Physical parameters of siltstone			
ρ	Density	2720 kg/m^3^	
φ	Friction angle	30°	
*n*	Porosity	5%	
Particle section			
$\frac{{R}_{max}}{{R}_{min}}$	Particle size ratio	1.66	
$\upmu$	Friction coefficient	–	
${ E}_{c}$	Effective modulus	–	${E}_{c}={\overline{E} }_{c}$
$\frac{{k}_{n}}{{k}_{S}}$	Normal-to-tangential stiffness ratio	–	$\frac{{k}_{n}}{{k}_{S}}=\frac{{\overline{k} }_{n}}{{\overline{k} }_{s}}$
Parallel bonding section			
${\tilde{\sigma }}_{c}$	Normal strength	–	
${\overline{ \tau } }_{c}$	Tangential strength	–	
${\overline{ E} }_{c}$	Effective modulus	–	${\overline{E} }_{c}{=E}_{c}$
$\frac{{ \overline{k} }_{n}}{{\overline{k} }_{s}}$	Normal-to-tangential stiffness ratio	–	$\frac{{\overline{k} }_{n}}{{\overline{k} }_{s}}=\frac{{k}_{n}}{{k}_{S}}$
${\overline{ \lambda } }_{n}$	Radius multiplier	1	

Referring to the results of previous research^2,19,20^, the particle effective modulus ${E}_{c}$ and parallel bonding effective modulus ${\overline{E} }_{c}$, the particle normal to tangential stiffness ratio ${k}_{n}/{k}_{S}$ and the parallel bonding normal to tangential stiffness ratio ${\overline{k} }_{n}/{\overline{k} }_{s}$ are taken to be the same (for the convenience of later discussion, the particle effective modulus ${E}_{c}$ and parallel bonding effective modulus ${\overline{E} }_{c}$ are unified by ${E}_{c}$, the particle normal to tangential stiffness ratio ${k}_{n}/{k}_{S}$ and the parallel bonding normal to tangential stiffness ratio ${\overline{k} }_{n}/{\overline{k} }_{s}$ are taken to be the same),and the parallel bonding radius coefficient ${\overline{\lambda }}_{n}$ is taken as 1.0^21^.

**Figure 3:**
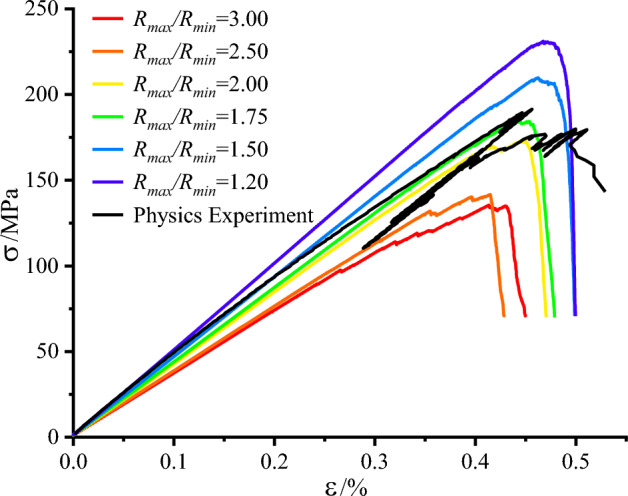
Stress–strain curves under different particle size ratios.

This paper investigates the optimal particle size ratio applicable to this study based on the control variable approach, and the minimum particle size of 1.5 mm is controlled, and uniaxial compressive numerical simulation experiments with ${R}_{max}/{R}_{min}$ ratios of 1, 1.25, 1.5, 1.75, 2, 2.5, and 3 are conducted in turn. The results show that the numerical simulation results are closest to the physical experiments when the particle size ratio is between 1.50 and 1.75, and the specific numerical simulation experimental results are shown in Fig. 3 below. This result is consistent with the previous proposal^2^ that ${R}_{max}/{R}_{min}$ is 1.66, so the particle size ratio in this paper is 1.66.

The number and size of particles have some influence on the nature of the macro-mechanical parameters of the model, therefore, it is necessary to consider the influence of the particle number *Res* (resolution) on the calculation results of macroscopic mechanical parameters at the smallest scale of the model. The previous work^22^ defined the *Res* as follows, and pointed out that when *Res* ≥ 10, the influence of particle number and size on the macro-mechanical parameters of the model is small. In this paper, the *Res* of the 50 mm diameter specimens used in the subsequent numerical simulations is greater than or equal to 10.

3 $$Res=\frac{L}{{R}_{min}}\times \frac{1}{1+\left({R}_{max}/{R}_{min}\right)}$$

where: *L* is the minimum scale of the model; ${R}_{max}$ is the maximum particle diameter; ${R}_{min}$ is the minimum particle diameter.

## Analysis of the correlation between macroscopic and microscopic parameters

The nonlinear mapping relationship between macro-mechanical parameters and micro-mechanical parameters is extremely complex, and multiple micro-mechanical parameters jointly determine and interact with each other to influence macro-mechanical parameters. In order to study the relationship between macro and microscopic parameters, a systematic analysis is needed to analyze the sensitivity of microscopic parameters to macro parameters. At present, most of the studies use mathematical and statistical methods, which are difficult to satisfy in systemic studies due to the high requirements of the amount of data and whether the data have obvious patterns. Based on this, this paper makes use of gray absolute correlation analysis, which has been widely used in various fields of socio-economic, engineering and natural sciences^23,24^. The basic idea is to determine whether the connection between different sequences is strong or not based on the area between different sequence curves. The basic theory of gray absolute correlation analysis^25^ is as follows:

Notation of the system behavior sequence as: *X*_*i*_ = (*x*_*i*_(1),*x*_*i*_(2),…,*x*_*i*_(n)), and write the line (*x*_*i*_(1)- *x*_*i*_(1), *x*_*i*_(2)- *x*_*i*_(1),…,*x*_*i*_(n)- *x*_*i*_(1)) as *X*_*i*_-*x*_*i*_(1). Let ${s}_{i}={\int }_{1}^{n}({X}_{i}-{x}_{i}\left(1\right))\mathrm{dt}$. Similarly, ${s}_{j}={\int }_{1}^{n}({X}_{j}-{x}_{j}\left(1\right))\mathrm{dt}$, then the gray absolute correlation between *X*_*i*_ and *X*_*j*_ is ${\varepsilon }_{ij}=\frac{1+\left|{s}_{i}\right|+\left|{s}_{j}\right|}{1+\left|{s}_{i}\right|+\left|{s}_{j}\right|+\left|{s}_{i}-{s}_{j}\right|}$ and $0<{\varepsilon }_{ij}\le 1$, The closer the absolute gray correlation ${\varepsilon }_{ij}$ is to 0 indicates that the correlation between the two system behavior sequences is smaller, and the closer it is to 1 indicates that the correlation between the two system behavior sequences is larger. ${\varepsilon }_{ij}\in \left(0,\left.1\right]\right.$ shows that no two behavioral sequences in the system can be strictly uncorrelated and that there are behavioral sequences that are absolutely correlated with each other.

Based on the simplified results of the microscopic parameters seven microscopic parameters were formulated for eight sets of numerical simulation tests. The numerical simulation tests include uniaxial compression and Brazilian splitting, using PFC software. After post-processing the test results to obtain the macroscopic parameters elastic modulus *E*, Poisson's ratio *ν*, compressive strength *σ*_*c*_ and tensile strength *σ*_*t*_, the specific parameters are shown in Table 2 below.

**Table 2:** Macro–micro parameters statistics.

Macro–Micro parameters	Lab 1	Lab 2	Lab 3	Lab 4	Lab 5	Lab 6	Lab 7	Lab 8
Micro								
${\overline{ E} }_{c}{=E}_{c}$(GPa)	10	15	20	25	30	35	40	45
$\frac{{\overline{k} }_{n}}{{\overline{k} }_{s}}=\frac{{k}_{n}}{{k}_{S}}$	0.5	0.55	0.6	0.65	0.7	0.75	0.8	0.85
$\upmu$	0.41	0.42	0.43	0.44	0.45	0.46	0.47	0.48
${\tilde{\sigma }}_{c}$(MPa)	75	80	85	90	95	100	105	110
${\overline{ \tau } }_{c}$(MPa)	100	150	200	250	300	350	400	450
Macro								
*E *(GPa)	11.405	16.449	21.446	26.135	30.663	36.067	38.268	43.154
*ν*	0.1494	0.1614	0.2412	0.3703	0.3243	0.3150	0.3067	0.1896
σ_c_ (MPa)	78.911	118.68	141.15	162.58	188.03	188.06	176.09	207.57
σ_t_ (MPa)	13.806	11.269	11.410	13.325	14.405	15.347	13.146	12.783

With the results of numerical simulation tests, the gray absolute correlation between seven microscopic parameters and four macroscopic parameters are calculated respectively, and a total of 20 Gy absolute correlation analyses are performed. Therefore, the solving process of the gray absolute correlation degree is discussed in detail by solving the correlation degree between the effective modulus and the elastic modulus as an example, and the solving process is as follows:

Firstly, the effective modulus is written in serial form

4 $$X_{0} = \left( {x_{0} \left( {1} \right),x_{0} \left( {2} \right), \ldots ,x_{0} \left( {8} \right)} \right) = \left( {{1}.00{\text{E}} + {1}0,{1}.{5}0{\text{E}} + {1}0, \ldots ,{4}.{5}0{\text{E}} + {1}} \right)$$

Similarly, the sequence of elastic moduli takes the form

5 $$X_{{1}} = (x_{{1}} \left( {1} \right),x_{{1}} \left( {2} \right), \ldots ,x_{{1}} \left( {8} \right) = \left( {{1}.{14}0{\text{5E}} + {1}0,{ 1}.{\text{6449E}} + {1}0, \ldots ,{ 4}.{\text{3154E}} + {1}0} \right)$$

Since both *X*_0_ and *X*_1_ are 1-time-distance sequences, there is no need to collate them. Secondly, the sequence *X*_0_ and *X*_1_ are then zeroed at the start point to obtain

6 $$X_{0}^{0} = \left( {x_{0}^{0} \left( 1 \right),x_{0}^{0} \left( 2 \right), \ldots ,x_{0}^{0} \left( 8 \right)} \right) = \left( {x_{0} \left( 1 \right) - x_{0} \left( 1 \right),x_{0} \left( 2 \right) - x_{0} \left( 1 \right), \ldots ,x_{0} \left( 8 \right) - x_{0} \left( 1 \right)} \right) = \left( {0,5000000000, \ldots ,35000000000} \right)$$

7 $$X_{1}^{0} = \left( {x_{1}^{0} \left( 1 \right),x_{1}^{0} \left( 2 \right), \ldots ,x_{1}^{0} \left( 8 \right)} \right) = \left( {x_{1} \left( 1 \right) - x_{1} \left( 1 \right),x_{1} \left( 2 \right) - x_{1} \left( 1 \right), \ldots ,x_{1} \left( 8 \right) - x_{1} \left( 1 \right)} \right) = \left( {0,5044000000, \ldots ,31749000000} \right)$$

Thirdly, compute $\left|{s}_{0}\right|$, $\left|{s}_{1}\right|$ and $\left|{s}_{1}-{s}_{0}\right|$, i.e.

8 $$\left|{s}_{0}\right|=\left|\sum_{k=2}^{n-1}{x}_{0}^{0}\left(k\right)+\frac{1}{2}{x}_{0}^{0}\left(n\right)\right|=\left|\sum_{k=2}^{7}{x}_{0}^{0}\left(k\right)+\frac{1}{2}{x}_{0}^{0}\left(8\right)\right|=122500000000$$

9 $$\left|{s}_{1}\right|=\left|\sum_{k=2}^{n-1}{x}_{1}^{0}\left(k\right)+\frac{1}{2}{x}_{1}^{0}\left(n\right)\right|=\left|\sum_{k=2}^{7}{x}_{1}^{0}\left(k\right)+\frac{1}{2}{x}_{1}^{0}\left(8\right)\right|=116472500000$$

10 $$\left|{s}_{1}-{s}_{0}\right|=6027500000$$

Finally, the grey absolute correlation between the effective modulus and the elastic modulus is calculated

11 $${\varepsilon }_{01}=\frac{1+\left|{s}_{0}\right|+\left|{s}_{1}\right|}{1+\left|{s}_{0}\right|+\left|{s}_{1}\right|+\left|{s}_{1}-{s}_{0}\right|}=0.9754$$

The results of the gray absolute correlation analysis of macro and micro parameters are shown in Table 3 below, and the results show that the microscopic parameters affecting the elastic modulus are mainly the effective modulus; the microscopic parameters affecting the Poisson's ratio are mainly the normal to tangential stiffness ratio and the friction coefficient, and ${k}_{n}/{k}_{S}>\upmu$; the microscopic parameters affecting the compressive strength are mainly. The microscopic parameters affecting the compressive strength are parallel bonding tangential strength, parallel bonding normal strength and effective modulus, and ${\overline{\tau }}_{c}$>$\overline{{\sigma }_{c}}$>*E*_*c*_; the microscopic parameters affecting the tensile strength are parallel bonding normal strength and parallel bonding tangential strength, and $\overline{{\sigma }_{c}}$>${\overline{\tau }}_{c}$

**Table 3:** Gray absolute correlation of macro–micro parameters.

Correlation of macro–micro parameters	Microscopic parameter				
	${E}_{c}$	${k}_{n}/{k}_{s}$	μ	$\overline{{\sigma }_{c}}$	${\overline{\tau }}_{c}$
Macroscopic parameters					
σ_*c*_	0.5022	0.5000	0.5000	0.6128	0.7216
σ_*t*_	0.5000	0.5000	0.5000	0.5181	0.5018
*E*	0.9754	0.5000	0.5000	0.5005	0.5053
Ν	0.5000	0.8891	0.7775	0.5000	0.5000

## Multifactor regression analysis of macro–micro parameters

Based on the gray absolute correlation analysis of macroscopic parameters and microscopic parameters, several numerical simulation tests were done. Afterwards, multiple regression was carried out through the data analysis software IBM SPSS Statistics to construct the mapping expression between macroscopic parameters and microscopic parameters of the PBM model. However, if the conventional experimental design is followed, the number of experiments and the complexity of experimental implementation lead to a large error in the multiple regression analysis. In this paper, we apply the experimental design method with high efficiency, rapidity and economy—orthogonal experimental design—in the regression analysis of macro–micro parameters.

### Multiple regression analysis of compressive strength

The microscopic parameters with greater correlation with compressive strength are parallel bonding tangential strength, parallel bonding normal strength and effective modulus, so a three-level, three-factor orthogonal test was designed with a total of nine number of numerical simulations, and its orthogonal table is shown in Table 4. The other parameters of the nine sets of numerical simulation tests were assigned the same values according to Table 5**,** changing only the three microscopic parameters mentioned above. After the numerical simulation of the nine sets of test data by the discrete element software PFC, the compressive strength of each set of numerical simulation tests was recorded, and then the multiple regression was performed by using the data analysis software IBM SPSS Statistics. The regression equation for the compressive strength regression analysis was

12 $${\text{s}}_{{\text{c}}} = \, 0.{583}\overline{\sigma }_{c} + 0.{465}\overline{\tau }_{c} - {1}.{554}$$

**Table 4:** Orthogonal table for regression analysis of compressive strength.

Lab	${\overline{\tau }}_{c}$(*MPa*)	$\overline{{\sigma }_{c}}$ (*MPa*)	*E*_*c*_ (*GPa*)	σ_c_ (*MPa*)
1	100	70	20	90.881
2	150	70	40	118.34
3	200	70	30	131.66
4	100	80	40	93.774
5	150	80	30	127.51
6	200	80	20	137.88
7	100	90	30	97.482
8	150	90	20	126.20
9	200	90	40	152.18

**Table 5:** Numerical model parameter.

R/mm	H/mm	n	ρ/kg•m^−3^	$\frac{{\mathrm{R}}_{\mathrm{max}}}{{\mathrm{R}}_{\mathrm{min}}}$	$\upmu$	$\mathrm{\varphi }$	$\frac{{\mathrm{k}}_{\mathrm{n}}}{{\mathrm{k}}_{\mathrm{s}}}$
25	100	5%	2720	1.66/1	0.5	30°	0.85

The regression coefficient R^2^ = 0.955, which indicates that the fit is good and can reflect the interrelationship of macroscopic microscopic parameters more accurately. It should be pointed out that the regression equation does not involve the effective modulus as a microscopic parameter, and the compressive strength regression equation is mainly controlled by the normal strength of parallel bonding and the tangential strength of parallel bonding. This result is caused by the magnitude of the correlation, and the microscopic parameters affecting the compressive strength are ranked in the order of their correlation as ${\overline{\tau }}_{c}$>$\overline{{\sigma }_{c}}$>${E}_{c}$, which further indicates that the effective modulus has a small enough effect on the compressive strength to be negligible.

As it has been shown above that the effective modulus has a small effect on the compressive strength, only the effect of parallel bonding normal strength and parallel bonding tangential strength on the compressive strength is discussed here.

**Figure 4:**
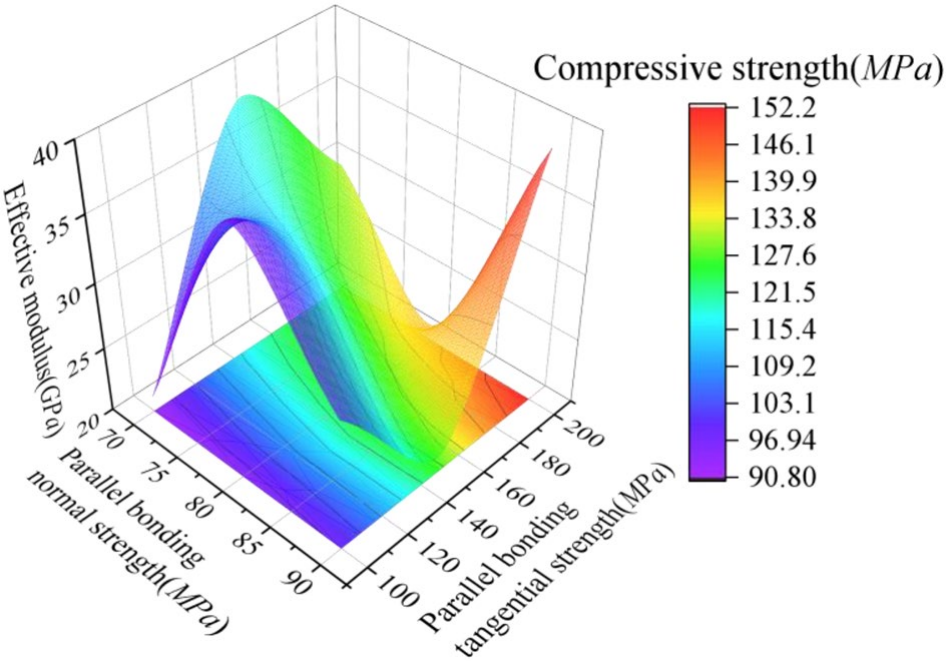
Mapping relationship between microscopic parameters and compressive strength.

In order to investigate the effect of interaction of single and multiple microscopic parameters on the compressive strength, the statistical data in Table 3 were processed to obtain Fig. 4. Considering the effect of individual microscopic parameters on compressive strength: keeping the parallel bond normal strength constant, the compressive strength increases with the parallel bond tangential strength. The correlation between compressive strength and tangential strength of parallel bonding remains constant if the normal strength of parallel bonding is gradually increased. However, the span of compressive strength increment interval gradually increases and the increment of compressive strength gradually tends to be flat. It indicates that the compressive strength is proportional to the parallel bonding tangential strength, but the sensitivity of the compressive strength to the parallel bonding tangential strength increases with the increase of the parallel bonding normal strength; Considering the effect of multiple microscopic parameters interaction on compressive strength: controlling the effective modulus and increasing both parallel bond tangential strength and parallel bond normal strength, the compressive strength increases with the increase of both. It shows that the compressive strength is positively correlated with the interaction of the two microscopic parameters.

### Multiple regression analysis of tensile strength

The microscopic parameters affecting the tensile strength are mainly parallel bond normal strength and parallel bond tangential strength, therefore, a four-level two-factor orthogonal test was designed, totaling 16 times the number of simulated tests, and the orthogonal table is shown in Table 6 below. The specific steps are the same as in section "Multiple regression analysis of compressive strength", and the numerical model parameters, except for the effective modulus of 45 GPa, are assigned in Table 4, and the regression equation for the multiple regression analysis of tensile strength is

13 $$\sigma_{t} = 0.{2}0{2}\overline{\sigma }_{c} + 0.0{26}\overline{\tau }_{c} - {8}.{449}$$

**Table 6:** Orthogonal table for regression analysis of tensile strength.

Lab	$\overline{{\sigma }_{c}}$ (*MPa*)	${\overline{\tau }}_{c}$(*MPa*)	σ_t_ (*MPa*)
1	70	100	6.8760
2	70	150	11.3587
3	70	200	10.5039
4	70	250	10.5038
5	80	100	9.8375
6	80	150	13.0742
7	80	200	14.7430
8	80	250	14.0778
9	90	100	12.2276
10	90	150	11.5246
11	90	200	15.1830
12	90	250	15.9233
13	100	100	14.3357
14	100	150	15.7146
15	100	200	17.1426
16	100	250	17.9007

The regression coefficient R^2^ = 0.861 indicates that the regression equation can better respond to the mapping relationship between the normal/tangential strength of parallel bonding and the tensile strength.

**Figure 5:**
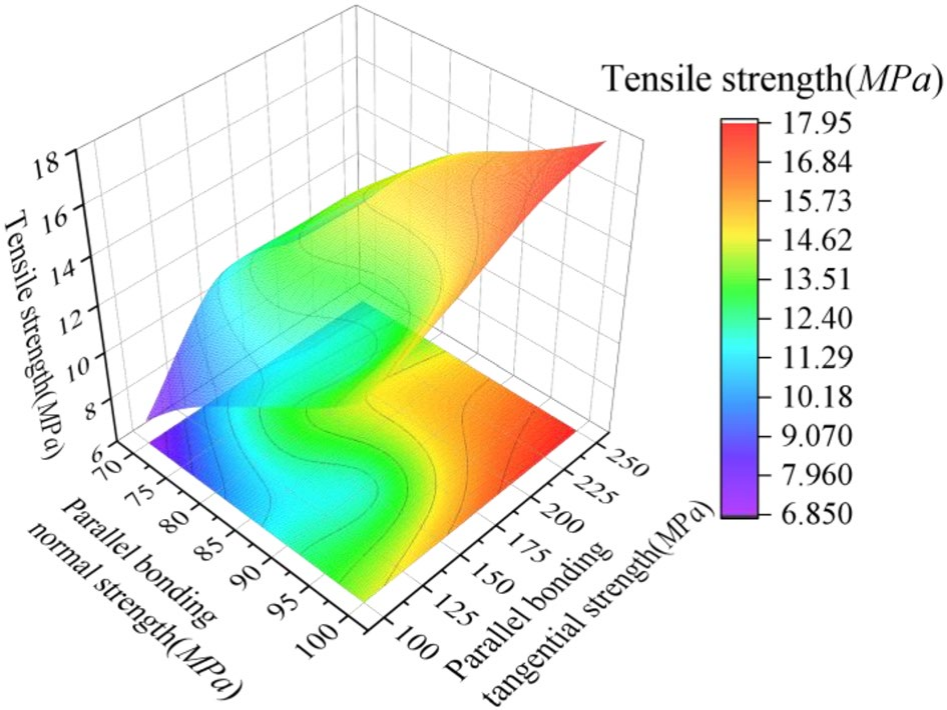
Mapping relationship between microscopic parameters and tensile strength.

When considering the effect of individual microscopic parameters on the tensile strength: keeping the tangential strength of parallel bonding constant, the tensile strength increases with the increase of the normal strength of parallel bonding. If the tangential strength of parallel bonding is gradually increased, the correlation between the tensile strength and the normal strength of parallel bonding is maintained, but the tensile strength is unevenly increasing and the beginning and end values do not show a good correlation. This indicates that the main influence on the tensile strength is the normal strength of parallel bonding. The influence of the interaction between the two on the tensile strength is considered: increasing or decreasing the parallel bonding normal strength and parallel bonding tangential strength at the same time, the tensile strength also increases or decreases. It is shown that the tensile strength is positively correlated with the interaction between the two. The details of the nonlinear mapping relationship between the microscopic parameters and the tensile strength are shown in Fig. 5 below.

### Modulus of elasticity/Poisson's ratio nonlinear regression analysis

The correlation between the microscopic parameters and the elastic modulus is ${E}_{c}>{\overline{\tau }}_{c}>\overline{{\sigma }_{c}}>{k}_{n}/{k}_{s}=\upmu$, then the effective modulus as the main factor affecting the elastic modulus; and the correlation between Poisson's ratio and the microscopic parameters is ${{k}_{n}/{k}_{s}>\upmu >E}_{c}=\overline{{\sigma }_{c}}$, which mainly affects Poisson's ratio The microscopic parameters in order are stiffness ratio and friction coefficient, but the correlation between them is large and the correlation between stiffness ratio and Poisson's ratio is close to 1, indicating that Poisson's ratio is mainly controlled by stiffness ratio. If both of them are included in the Poisson's ratio regression analysis, it will greatly increase the number of tests, so the friction coefficient is not considered. The main material parameters of the numerical simulation are shown in Table 4, and the rest of the material parameters are assigned to the data in Table 7 below. According to the test procedure in Section "Multiple regression analysis of compressive strength", the regression equations between the macroscopic parameters are as follows, and the regression coefficients R^2^ are 0.959 and 0.967 respectively, indicating that the regression equations can better reflect the relationships between the elastic modulus and effective modulus, and Poisson's ratio and stiffness ratio.

**Table 7:** Elastic modulus/poisson's ratio regression analysis table.

Lab	1	2	3	4	5	6	7	8	9
*E*_*c*_ (*GPa*)	20	25	30	35	40	45	50	55	60
*E* (*GPa*)	9.7611	24.7340	29.4692	34.6243	38.7721	43.7155	48.9284	53.4759	58.2904
Lab	10	11	12	13	14	15	16	17	18
${k}_{n}/{k}_{s}$	1.0	1.5	2.0	2.5	3.0	3.5	4.0	4.5	5.0
ν	0.10551	0.15961	0.19534	0.21739	0.23776	0.25403	0.26845	0.27944	0.28845

14 $$\left\{\begin{array}{c}E =0.595{E}_{c}1.126 \\ \nu =0.128\left({k}_{n}/{k}_{s}\right)0.529\end{array}\right.$$

**Figure 6:**
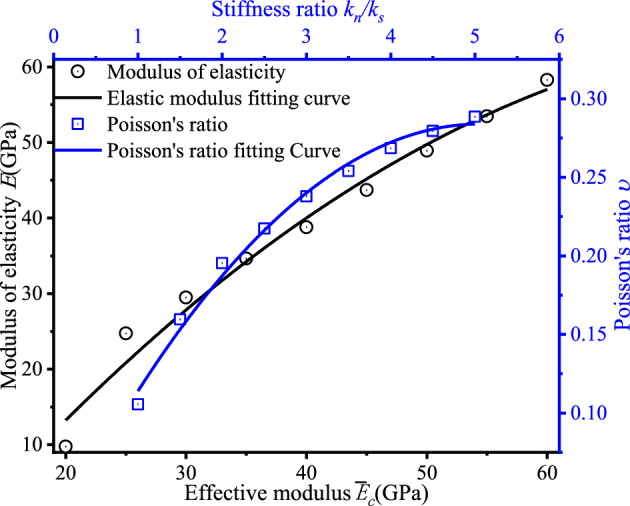
$${E}_{c}-E,{k}_{n}/{k}_{s}-$$ ν.

To further demonstrate the mapping relationship between elastic modulus and effective modulus, Poisson's ratio and stiffness ratio, the data in the above table were post-processed to obtain the following Fig. 6. The elastic modulus increases with the stiffness ratio, but the growth rate of the elastic modulus decreases as the stiffness ratio increases gradually. The Poisson's ratio is affected by the stiffness ratio in the same way as the former. It shows that the two macroscopic parameters, effective modulus and elastic modulus, and stiffness ratio and Poisson's ratio, are positively correlated with each other, and the growth rate decreases as the corresponding microscopic parameters gradually increase.

In summary, the correlations between the macro and microscopic parameters were analyzed by the gray absolute correlation, based on which orthogonal tests and nonlinear regression analysis were performed to obtain the calibration equations for the microscopic parameters as shown in Table 8 below the correlation coefficients indicate that this regression equation can better reflect the nonlinear mapping relationship between the macro and microscopic parameters.

**Table 8:** Calibration equations for microscopic parameters.

Macro-parameters	Regression equation	Correlation coefficient
σ_c_	σ_c_ = 0.583 $\overline{{\sigma }_{c}}$+0.465 ${\overline{\tau }}_{c}$-1.554	*R*^2^ = 0.955
σ_t_	σ_t_ = 0.202 $\overline{{\sigma }_{c}}$+0.026 ${\overline{\tau }}_{c}$-8.449	*R*^2^ = 0.861
*E*	*E* = 0.595 ${E}_{c}$ ^1.126^	*R*^2^ = 0.959
*v*	ν = 0.128 $\left({k}_{n}/{k}_{s}\right)$^0.529^	*R*^2^ = 0.967

## Microscopic parameters calibration method validation

On the basis of the previous discussion, the macroscopic mechanical parameters of a group of siltstones were substituted into Table 8 to calculate the calibrated microscopic parameters, which are shown in Table 9 below. In order to avoid the dispersion brought by the macroscopic mechanical parameters obtained from a single set of physical experiments, the macroscopic parameters in Table 9 are obtained by taking the average value of 3 sets of specimens. And the method of Poisson's ratio *v* determination^26^ is shown in the following Eq. (15), where ${\varepsilon }_{d50}$ indicates the transverse strain value when the stress is 50% of the compressive strength, and ${\varepsilon }_{h50}$ indicates the longitudinal strain value when the stress is 50% of the compressive strength.

**Table 9:** Calibration values of microscopic parameters.

Siltstone macroscopic parameters			Microscopic parameters calibration value			Remark
Symbol	Unit	Value	Symbol	Unit	Value	
*n*	–	5%	*n*	–	5%	Same part
*ρ*	kg•m^−3^	2720	*ρ*	kg•m^−3^	2720	
*E*	GPa	48.92	${\overline{E} }_{c}{=E}_{c}$	GPa	50.19	Calibration part
σ_c_	MPa	191.76	${\tilde{\sigma }}_{c}$	MPa	108.9127	
σ_t_	MPa	20.81	${\overline{\tau }}_{c}$	MPa	279.1783	
ν	–	0.13	$\frac{{\overline{k} }_{n}}{{\overline{k} }_{s}}=\frac{{k}_{n}}{{k}_{S}}$	–	1.03	

15 $${\nu }_{50}=\frac{{\varepsilon }_{d50}}{{\varepsilon }_{h50}}$$

The comparison of the macroscopic mechanical parameters, stress–strain curves and damage modes obtained from the discrete element numerical simulation of siltstone with the experimental results of the finite element software ABAQUS is used to verify the reasonableness and correctness of the calibration method of the microscopic parameters in this paper.

### Discrete element numerical simulation of uniaxial compression/Brazilian splitting in siltstone

**Figure 7:**
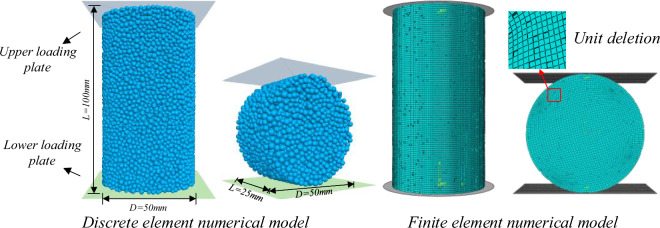
Uniaxial compression and Brazilian splitting numerical model.

The discrete element numerical model was established by relying on the requirements of SL264-2001 "Rock Test Procedure for Water Conservancy and Hydropower Engineering" for the specimen size^26^, which used a cylindrical specimen with a length (L) to diameter (D) ratio of 2 for the uniaxial compression test and a disc specimen with L/D of 0.5 for the Brazilian splitting test, the specific dimensions of which are shown in Fig. 7 below. The numerical simulation experiments were performed by the upper and lower loading plates were loaded relative to each other at a rate of 0.05 mm/s, and the loading was stopped when the specimen was completely damaged. The stresses, strains, fractures, and AE(acoustic emission) events inside the model were monitored during the test in order to analyze the damage pattern of the siltstone under compression and tensile loading, respectively.

**Figure 8:**
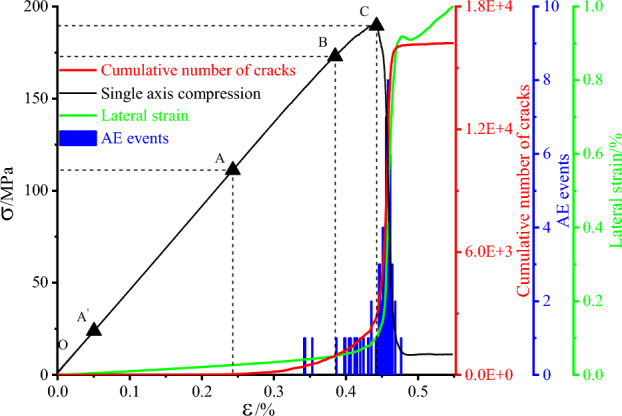
Monitoring curve of siltstone under uniaxial compression.

The results of the uniaxial compression numerical simulation tests are shown in Fig. 8 below, and the stress–strain curves are mainly divided into the pore compacting stage (O–A'), the elastic deformation to microelastic fracture stable development stage (A'–B), the unstable rupture development stage (B–C), and the post-rupture stage (after point C)^27^. The AE events were used to summarize these four stages. At the beginning of loading, this stage was mainly to close the pores inside the model because a porosity of 5% was set in the discrete element model. However, the curve in this stage does not show the traditional up-concave shape, but slightly oscillates. The reason for this is that the model parameters are taken from hard rock and the porosity is low, and no cracks are generated in this stage; the loading continues to the stage of elastic deformation and stable development of microelastic fractures, and the stress–strain curve in this stage is approximately linear. There are a few microcracks in the AB section, mainly in the contact area between the loading plate and the model, and the cumulative number of cracks also confirms this; as the loading plate continues to be loaded, the model enters the unstable rupture development stage, and the model changes from elastic to plastic. At this stage, the number of microcracks in the contact part of the loading plate and the model continues to increase and microcracks are generated in the middle of the model; finally, a large number of microcracks grow exponentially in the rupture stage, and the cracks cross and unite with each other to form a macroscopic fracture surface, and the stress drops rapidly, but not to 0. The specific damage mode is shown in Fig. 11 below, in which the black part represents the microcracks, and the model is mainly damaged by single bevel shear, but in the loading process The model is mainly in single-slope shear damage, but during the loading process, the loading plate has an "end effect" on the model, resulting in a large number of microcracks at the end of the model, which unite with the main crack to form a new crack.

**Figure 9:**
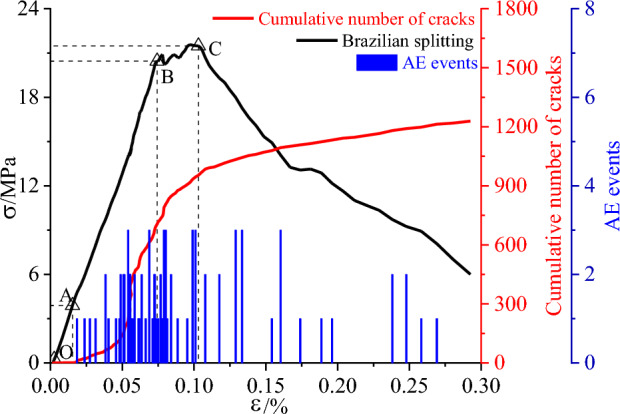
Monitoring curve of siltstone splitting in Brazil.

The results of the Brazilian numerical simulation test are shown in Fig. 9 below. The stress–strain curve of the Brazilian splitting test differs from that of the uniaxial compression test in that the fissures appear rapidly at the beginning of the load application (i.e., point A) and then grow linearly to point B. It should be noted that the fissures generated in the A-B section are mostly fissures generated at the contact area between the loading plate and the model; as the loading continues, the siltstone specimen enters the yielding stage (B–C section), where the fissures at the ends of the specimen continue to increase and tend to extend toward the middle of the specimen. The cracks at the end of this stage continue to increase and have a tendency to extend to the middle of the specimen; once the stress exceeds the tensile strength of the siltstone (after point C), the cracks at both ends of the siltstone specimen will rapidly extend inward and converge to form a tensile crack through the specimen, and the specific damage pattern is shown in Fig. 13 below.

The data in Figs. 8 and 9 were processed to obtain the macroscopic mechanical parameters of the siltstone, and compared with the macroscopic mechanical parameters obtained by physical tests on the siltstone, and the specific results are shown in Table 10 below the relative errors are within 10%, which indicates that the calibration method of the microscopic parameters is reasonable.

**Table 10:** Comparison of macroscopic mechanical parameters.

Macro parameters	Physical tests	Discrete element numerical simulation	Absolute error	Relative error (%)
σ_c_/MPa	191.76	189.4432	−2.3168	1.2
σ_t_/MPa	20.81	20.3025	−0.5075	2.4
*E*/GPa	48.92	45.1522	−3.7678	7.7
*v*	0.13	0.1178	−0.0122	9.4

### Comparison of finite element and discrete element numerical simulation results

The uniaxial compression and Brazilian splitting finite element numerical simulation tests of siltstone need to be consistent with the discrete element numerical simulation in terms of model size, parameter settings, loading method and loading speed, which is a prerequisite for comparison between the two. And in order to fit the reality, the unit deletion of the finite element numerical model is carried out by the self-programmed script. To simulate the siltstone with 5% porosity, the specific numerical model size is shown in Fig. 7 above.

**Figure 10:**
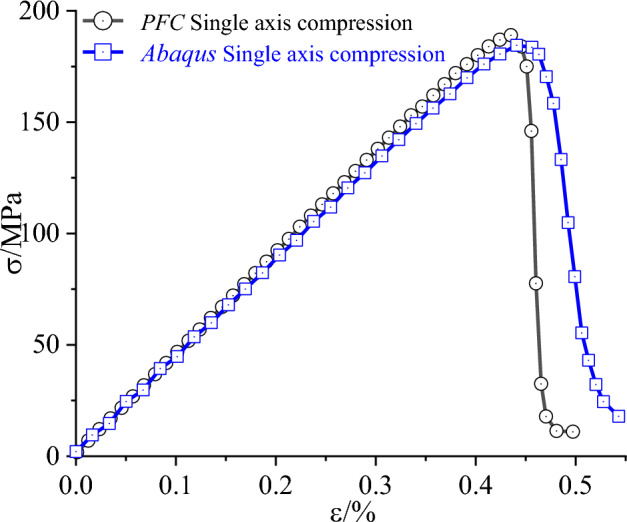
Uniaxial compression stress–strain curve of siltstone.

**Figure 11:**
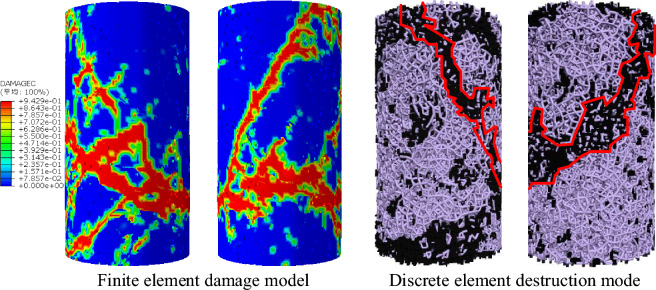
Failure mode of siltstone under uniaxial compression.

The stress–strain comparison between the finite element and discrete element uniaxial compression numerical simulation tests is shown in Fig. 10 below, and both curves are relatively similar; as far as their damage modes are concerned, they are both dominated by monoclinic shear damage, and the specific damage modes are shown in Fig. 11 below; it shows that the results of the discrete element uniaxial compression tests on chalk sandstone are valid using the microscopic parameters calibration method proposed in this paper.

**Figure 12:**
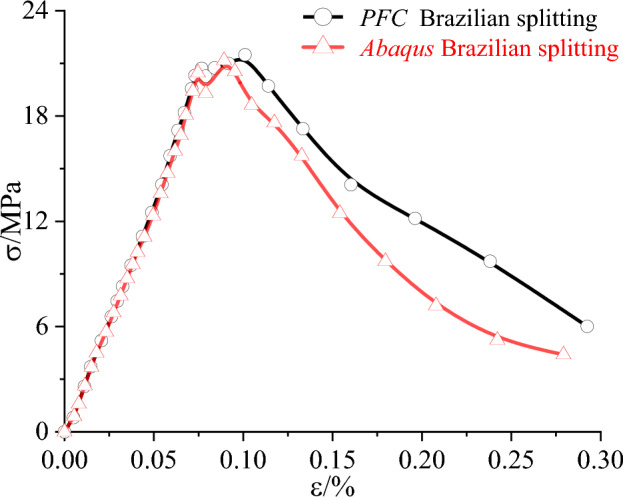
Stress–strain curve of Brazilian cleavage in siltstone.

**Figure 13:**
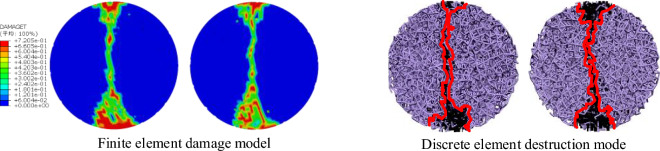
Brazilian fracture failure mode of siltstone.

The results of the discrete element numerical simulation tests of Brazilian splitting in siltstone are in high agreement with the results of the finite element numerical simulation tests both in the stress–strain curve (Fig. 12) and in the damage mode (Fig. 13), indicating that the results of the discrete element Brazilian splitting tests in siltstone using the microscopic parameters calibration method proposed in this paper are valid. In summary, it shows that the proposed microscopic parameters calibration method is reasonable and effective.

## Conclusion

This study explores the correlation between microscopic parameters and macroscopic parameters of the PBM model with the gray absolute correlation as the core. Single or multiple microscopic parameters interact with each other to jointly affect the macroscopic mechanical parameters. Among them, the parallel bond normal/tangential strength mainly affects the compressive and tensile strengths, and the parallel bond normal/tangential stiffness ratio and particle normal/tangential stiffness ratio mainly determine the Poisson's ratio. Parallel bond effective modulus and particle effective modulus mainly determine the elastic modulus and indirectly affect the compressive strength.

On this basis, the nonlinear mapping relationship between the two is constructed on the multivariate nonlinear regression analysis. The compressive strength is directly proportional to the tangential strength of parallel bond, but with the increase of the normal strength of parallel bond, the sensitivity of the compressive strength to the tangential strength of parallel bond increases, while the tensile strength is mainly controlled by the normal strength of parallel bond. The two sets of macroscopic parameters, elastic modulus and effective modulus, Poisson's ratio and stiffness ratio, are positively correlated with each other, but the growth rate of the macroscopic parameters is negatively correlated with them.

The rationality of the new method of microscopic parameters calibration is demonstrated by taking siltstone as an example and combining it with finite elements. The method can be generalized to the calibration of microscopic parameters of other intrinsic models, and the theoretical results can provide ideas for the calibration of microscopic parameters of the same type of rocks.

## Data Availability

All data generated or analysed during this study are included in this published article.

## Abbreviations

PFC: Particle flow code software
PBM: Linear parallel bond model
Res: Resolution
BP: Back propagation
AE: Acoustic emission
